# Net present value approaches for drug discovery

**DOI:** 10.1186/2193-1801-2-140

**Published:** 2013-04-01

**Authors:** Andreas M Svennebring, Jarl ES Wikberg

**Affiliations:** Department of Pharmaceutical Biosciences, Division of Pharmaceutical Bioinformatics, Biomedical Centre, Uppsala University, Box 591, SE751 24 Uppsala, Sweden

**Keywords:** Biotechnology, Drug development, Drug discovery, Investment under uncertainty, Life science, *NPV*, Risk-adjusted net present value, *rNPV*

## Abstract

Three dedicated approaches to the calculation of the risk-adjusted net present value (*rNPV*) in drug discovery projects under different assumptions are suggested. The probability of finding a candidate drug suitable for clinical development and the time to the initiation of the clinical development is assumed to be flexible in contrast to the previously used models. The *rNPV* of the post-discovery cash flows is calculated as the probability weighted average of the *rNPV* at each potential time of initiation of clinical development. Practical considerations how to set probability rates, in particular during the initiation and termination of a project is discussed.

## Background

Drug discovery and development programs offer particular difficulties in the estimation of profitability, largely due to their high attrition rates, but they also offer opportunities to handle the problem because of the well-defined phases of the process. Drug discovery and development programs constitute a special case of investment under ongoing uncertainty (Dixit and Pindyck 1994; Schwartz and Trigeorgis 2004). The current main approach for evaluation is modifications of the classical net present value concept; in particular, the risk-adjusted net present value (*rNPV*) method is commonly employed, according to which the project value is determined as:1

where *C*_*n*_ is the *n*^th^ cash flow of *N* in total, *R*_*0*_ and *R*_*n*_ is the estimated probability of obtaining the entire series of cash flows from the initiation of the project and from the *n*^th^ cash flow, respectively, *r* is the discount rate and *t(n)* the time of the *n*^th^ cash flow (Stewart 2002; Stewart et al. 2001). Presentations and demonstrations of the implementation of the *rNPV* calculation in drug development projects found in literature generally start only right at the initiation of the phase I clinical trial, in the discovery and development pipeline. The complexity of the drug discovery, the high costs for producing suitable data, and the limited access to such information for actors outside organizations undertaking discovery activities, it may seem difficult to model the cost of the drug discovery process. In projects requiring major economical investments such as the construction of a building, bridge, oil platform or the clinical development of a drug, the time required for each development step is uncertain. While a clinical phase III trial usually takes 3–5 years, the average of 4 years may be a good estimate for the *rNPV* approach. However, in drug discovery endeavors, the time from initiation of the project to the generation of the first candidate drug ready for clinical development may vary from a few years and up without any distinct upper limit or guarantee that a compound ever will be found.

Nevertheless, in view of the high risk involved and the constant decline in productivity among the industry, the financial aspects of drug discovery endeavors, *rNPV* extensions tailored for the evaluation of drug discovery projects are urgently needed. Herein, a few approaches valid under different assumptions are suggested and discussed.

### Model framework

We assume that the total risk-adjusted net present value (*rNPV*_*tot.*_) of the expected cash flows resulting from a drug discovery endeavor is the value of the cash flows during the discovery phase (*rNPV*_*D*_) and the real value of the cash flows post-discovery (*rNPV*_*PD*_), hence:2

The problem with the valuation of the post-discovery phase is that the time of its initiation is unknown, if even it is ever to arrive. To simplify handling the valuation of the post discovery cash flows, we define the *time dependant post-discovery net present value* (*rNPV*_*PD, t*_) as the *rNPV* that we would face assuming that the development was initiated at time *t*. We also define the *current post-discovery net present value* (*rNPV*_*PD, 0*_) as the *rNPV* of the post-discovery cash flows assuming that the development start at present time, *i.e.* when the phase I clinical trial is just about to begin. In the methods described here, it is assumed that the valuation of the post-discovery cash flows is done as a separate entity through the *rNPV* method. The *rNPV*_*PD, t*_ can thus be calculated at a specific time (*t*) from the *rNPV*_*PD, 0*_:3

The *rNPV*_*D*_ is calculated as the sum of the products between the probability that a scenario (the *n*^th^ scenario) takes place (*p*_*n*_), and the *NPV* that would follow from that scenario:4

In the same vein, the *rNPV*_*PD.*_ is calculated as the sum of the products between the probability that the *n*^th^ scenario takes place (*p*_*n*_), and the *rNPV* that would follow from that scenario:5

### New model suggestions

Instead of dividing the discovery phase in parts, the process is seen as a black box generating compounds ready for clinical development with a certain probability. The planned time cause of the discovery process is divided in periods of constant length, which is set on a pragmatic basis, and the project continuous for *N* time periods. The chance of finding a compound ready for clinical development is either constant (*p*) or different (*p*_*n*_) between the different time periods.

The ability of a research organization to generate compounds fit for clinical development must be highly related to the economic resources allocated to the organization. We therefore assume that under the use of a fixed set of technologies the probability (*p*_*n*_) of finding a compound as the result of an investment is proportional to the product of the cash flow allocated to discovery research activities at the time interval and a constant (*P*) denoting the probability of finding a compound per monetary unit:6

where *C*_*n*_ denotes the cash flow to discovery activities at the *n*^th^ interval.

In many drug discovery projects, the synthesis and early ADMET (absorbtion, distribution, metabolism, elimination and toxicology) investigations are done by contract research organizations (CROs). When a compound suited for clinical developments has been found the entire focus may swiftly shift to the clinical phase. Our first model is based on the assumption that a drug discovery project is run until a suitable candidate for clinical development has been found, at which time point the discovery activities cease and the clinical development is initiated. The project goes on for a finite period of time of totally *N* periods.

**Figure 1:**
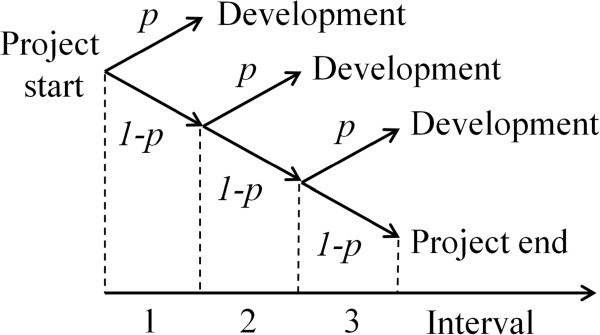
**Probability tree diagram showing possible scenarios for finding a compound fit for development with their associated probabilities indicated above and under the arrows.** The probability expressions are not cumulative. During the first period, the probability of generating a candidate drug is *p*, while the probability of not generating one is 1-*p*. If no drug is discovered during the first interval, a new chance is given during a second interval, which also has a probability of *p* for success. If no candidate drug is found during the first two periods, a last chance is given during the third interval.

In our first model (Figure 1) we further assume that the probability of finding a compound for development is constant during the entire discovery period as a result of a fairly constant cash flow to the research activities over time. During the first time period, the probability of finding a candidate drug is *p*. If no candidate is found during this period, the cumulative probability of finding a candidate in the second period is consequently (1-*p*)*p*, in the third it is (1-*p*)^2^*p*, and after *n* time periods it is (1-*p*)^*n-1*^*p*. The effective *rNPV* experienced if a candidate drug is discovered during time period *n* is therefore (1-*p*)^*n-1*^*p*∙*NPV*_*PD, t(n)*_. The *rNPV* post discovery is consequently:7

In accordance with (7), the *rNPV*_*D*_ is calculated as:8

**Figure 2:**
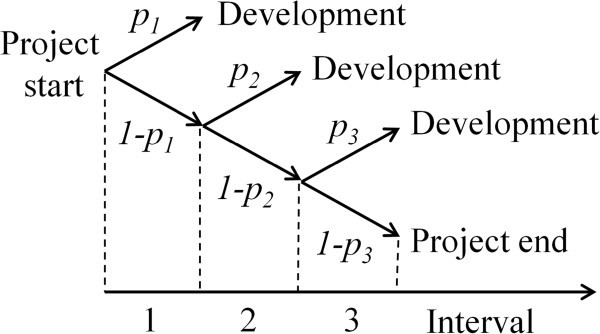
**Probability tree diagram showing the possible scenarios in a drug discovery project conveying to Figure**1**, where the probabilities for success are different for the different periods.**

In cases where the cash flow to the discovery activities is changing considerably over time, it might be necessary to use different probability rates at different time intervals. In our second model (Figure 2), we assume that the probability of finding a candidate drug for further development is *p*_*n*_ for the *n*^th^ period. During the first period, the probability of finding a candidate drug for development is thus *p*_1_, during the second period it is *p*_2_, leading to a cumulative probability at *t* = 0 of (1-*p*_1_)*p*_2_, and accordingly at the third interval to (1-*p*_1_)(1-*p*_2_)*p*_3_. The cumulative probability of finding a candidate at the *n*^th^ interval is consequently: (1-*p*_1_)(1-*p*_2_)… (1-*p*_*n*-1_)*p*_*n*_, *i.e.*:9

From this expression we can calculate the *rNPV*_*PD*_ as the sum of the probability that the development will be initiated in each possible time point multiplied with the *rNPV*_*PD, t(n)*_ of that particular time point:10

and accordingly the *rNPV*_*D*_:11

In our third model, we assume that more than one compound may be selected for clinical development. We also assume that the project continues even in the event that a candidate compound is found. If a large number of compounds are prepared and the probability that one of the compounds will be suitable as a drug is *p*, the chance of finding two compounds during the same time period is *p*^2^, and the chance of finding three is *p*^3^. The probability of finding *m* compounds for further development at one time interval is thus *p*^*m*^. The model is designed particularly for application in projects where the probability of finding compounds for development is high enough to make it probable to find more than one suitable compound for development during the same time period, and where it is judged meaningful to initiate the clinical development with multiple compounds.

Apparently, there is a limit how many compounds that can be prepared in a project and consequently how many compounds that can be found for further development. Strictly speaking, if *A* compounds are prepared in a project, the probability of finding a second compound for development would be *p*(*A*-1)/*A*, the third compound *p*(*A*-2)/*A*, and so on. However, due to the extremely large number of compounds theoretically possible to prepare in a drug discovery endeavor, we assume that the probability of finding additional compounds for development is equally high. Since the probability of finding multiple compounds quickly decreases with increasing exponent, we suggest that no upper limit is set for how many compounds that may be found fit for development in order to render a generally accepted expression. Under these assumptions, the average amount of compounds under development equals the sum of the product between *m* and *p*^*m*^:12

The *rNPV* of one time interval equals the average amount of compounds for development for the interval multiplied with the *rNPV*_*PD, t*_ at this time:13

and for the entire time period during which discovery activities are planned spanning *N* time intervals, the *rNPV*_*PD*_ and *rNPV*_*D*_ equals:14

and15

respectively.

We wish to demonstrate the utility of the model from a few simple calculations in which rough estimates of the parameters have been deduced from publicly available data. It has been estimated that the discovery phase account for approximately 30% of the total discovery and development cost of a drug, which may be around $1b making $300 m a rough estimate of the outgoing cash-flow necessary for the discovery of a drug including failing compounds (DiMasi et al. 2003). Of the drugs entering into phase I trials, approximately 5% later reach approval by regulatory authorities (Arrowsmith 2012). The cost per compound entering clinical development is thus $15 m and *P* = 1/$15 m approximating 7 10^-8^ compounds/$.

**Figure 3:**
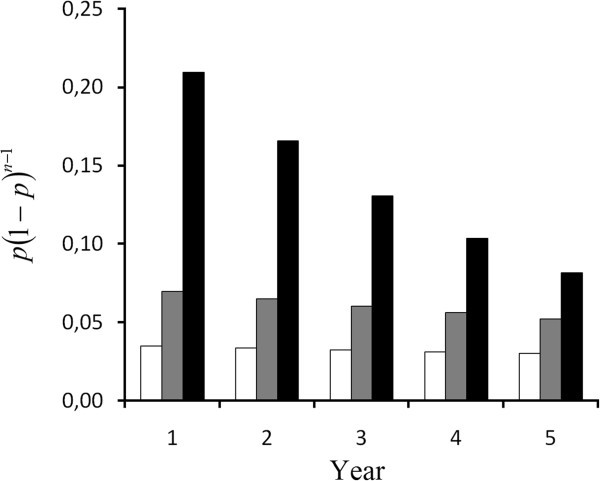
**The probability of finding a drug in year 1–5 with a probability rate*P*of 7 10**^**-8**^**compounds/$ and a constant cash-flow to the discovery activities of $0.5 m (empty bar), $1 m (gray bar) and $3 m (black bar) according to model 1.**

Assume a drug discovery project which has reached a level of maturity with compounds in all different stages of preclinical development and where the same cash-flow is fed to the discovery activities each year. Under the assumptions in model 1 (and 2), the probability of finding the only compound to proceed with into clinical development during one particular year from start equals *p(1-p)*^*n-1*^. In contrast, the number of compounds for clinical development weighted with the probability of occurrence does not change over the years as apparent in equation 9. The probability of finding one compound (model 1) or the expected amount of compounds weighted against the probability (model 3) has been calculated for the project described in which an annual cash-flow to the discovery work is $0.5 m, $1 m or $3 m per year, respectively (Table 1). The data from model 1 has been graphically displayed in Figure 3.

**Table 1:** **The probability of finding a drug in year 1–5 according to model 1 and 3**

		Cash-flow to discovery/year (million $)		
	n	0,5	1	3
Model 1	1	0,0350	0,0700	0,2100
	2	0,0338	0,0651	0,1659
	3	0,0326	0,0605	0,1311
	4	0,0315	0,0563	0,1035
	5	0,0304	0,0524	0,0818
Model 3	All	0,0376	0,0809	0,3365

A probability rate *P* of 7 10^-8^ compounds/$ and a constant cash-flow to the discovery activities of $0.5 m, $1 m and $3 m per year has been used. In model 3, the probability weighted amount of drugs that may be found.

In model 1 with a cash-flow of $0.5 m/year, the chance of finding a compound is 3.5% and falling slightly over the coming years due to the chance that a compound has been found during the previous years and the discovery work will be discontinued. The decrease becomes higher with increasing annual allocation and with a cash-flow of $3 m/year, it is after four years half of the probability of the first year. While using model 3, the probability weighted amount of compounds found with an annual investment of $0.5 m is only slightly higher than what was found for model 1, an increase that is due to the fact that in model 1 only one compound can be found, but more than one in model 3. However, the probability of finding two compounds suitable for development with this limited budget is very low. With increasing annual allocations, the probability of finding a second compound starts to affect the probability rate considerably.

## Results and discussion

Mathematical models are fabrications designed to capture the most essential aspects of reality. It is therefore imperative to acknowledge all imperfections to the models and to the degree it is possible to account for them in the process of setting the parameters (*i.e.* parameter estimates) fed to the model or to craft the models. The probability of finding a compound fit for development is likely to change from the beginning of a project and onwards due to experience, and a considerable lag time must be expected between cash flows to the project and the probability that a compound fit for development will be generated as a result of the investment.

**Figure 4:**
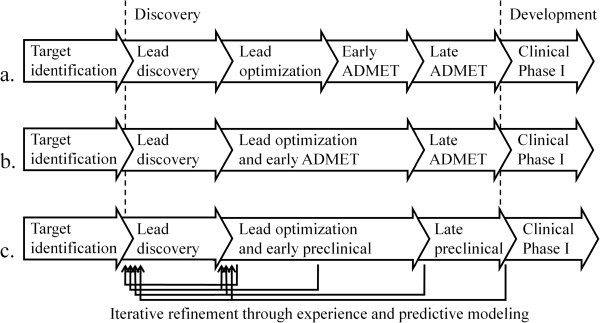
**Drug discovery starts after a target has been identified and ends with the initiation of clinical phase I, the later which is part of the drug development activities. a**. In the traditional Figure, the lead optimization process is focused on optimizing the functional activity of the compounds, after which the early preclinical phase commences. **b**. Today, lead optimization usually focuses not only on optimizing functional activity but also on the early ADMET properties. The lead optimization and early preclinical steps are therefore fused into one step. **c**. During the course of the discovery pipeline, data is generated describing the ability of the synthesized compounds to function as drugs. The data is utilized for the identification of new compounds for synthesis that are more likely than the previously made to excerpt the prerequisites to be acceptable for clinical drug usage.

The process of drug discovery can be divided into steps and functions in slightly different ways (Ashburn and Thor 2004; Pritchard et al. 2003; Schirle et al. 2012). We prefer to present the process without being too detailed in the description about what exact tasks and functions are done in each step since these often overlap in time, and we assume that the target identification and validation step is already finished at the onset of the discovery phase. The initiation of a drug discovery project is usually preceded with the identification of a target, such as a receptor protein; the function of which is going to be manipulated with a new drug (Figure 4).

**Figure 5:**
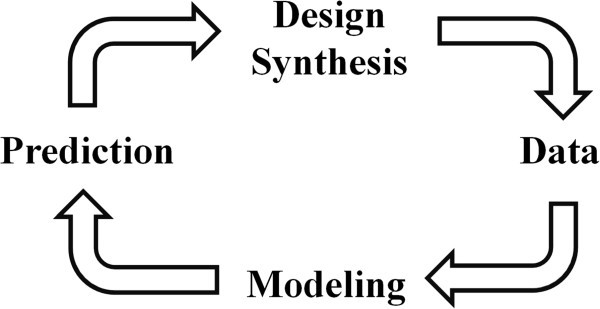
**The iterative refinement cycle through which experience is utilized to increase the probability of success in coming series of compounds.** Compounds are synthesized in a project and subjected to assays and animal tests, which generate data that indicate the likelihood for a compound to be functional as a drug. The data is used to create statistical mathematical models that describe the connection between chemical structure and expected behavior of the compounds. Such models may be directed to compound activities on multiple targets, to ADME properties and toxicity, and include approaches such as QSAR and proteochemometric modeling. The models may be based on the data developed within the project, as well as on public data and data from earlier projects. The model are used to predict the behavior of novel structures, based on which the most promising in the next series to be synthesized will be based on. Over time the models become more and more predictive when the process is iterated, which will increase the likelihood of finding suited compounds. The entire process is implemented on a broad systematic scale among the industry under the name ‘predictive modeling’.

The first step in the discovery phase is generally termed lead discovery and has the goal of identifying novel compounds with a weak drug effect, serving as starting points for further development. From the lead structure, a structural core is identified and the substituents, *i.e.* the “decoration” of the core, is tailored to render an optimal structure with respect to target activity and ADMET properties, This is a process known as lead optimization. The optimized compounds are subjected to a number of tests to investigate the faith of the compounds in the human body and their ability to cause toxic effects. These activities are often referred to as ADMET and are often divided in an early and a late preclinical phase, where the former primarily focuses on *in vitro* tests, and the latter is primarily concerned with animal tests. Today, the optimization process is usually intertwined with the early ADMET, since both ADMET qualities and drug activities are nowadays usually concomitantly concerned during the optimization process. During the course of a project, considerable experience is gained with time. With the synthesis of increasing amounts of compounds, it is possible to describe the connection between chemical structure and functional activity at the target, so called structure-activity relationships (SARs) that can guide the researchers into a more prosperous direction later in the project (Lewis 2005; Lin et al. 2006). Also for the ADMET properties, relationships between chemical structure and drug properties can be predicted based on previous experience (Ponec et al. 1999; Shen et al. 2003). Specifically, the data produced from physicochemical characterizations, biological *in vitro* assays and animal *in vivo* experiments on the accumulating compounds synthesized during the course of a project are used to create statistical mathematical models from which the performance of not yet synthesized compounds can be predicted (Figure 5) (Wikberg et al. 2011). With the advent of the models, new improved compounds can be hypothesized, synthesized and evaluated based on predictions of the models. The process is now implemented on a broad scale among the industry, under the name ’predictive modeling. The ability to generate compounds for clinical development is therefore likely to increase with time during the project.

When the models presented herein are used practically, it must be considered that the resource allocation between early and late activities in the discovery pipeline differ between projects, and in particular between newly founded and mature projects. Assume the life cycle from foundation to termination of a drug discovery enterprise based on only one single project, a so-called pure play company. In the newly founded enterprise, not so many compounds have qualified for preclinical investigations yet. In many cases, the entire outgoing cash flow goes to compound synthesis, *i.e.* discovery and optimization of leads. Maybe the lead optimization process has not even yet started. The probability of obtaining a compound fit for clinical development must be practically null under these circumstances. However, while the project or company matures, an increasing fraction of the research spending goes to preclinical activities. In this stage of maturity, the prospect of generating compounds fit for clinical development becomes considerable. When the drug discovery pipeline is well-populated with compounds, all four phases are cost drivers and the probability of generating a candidate drug is decent.

In view of the above discussion it is possible to utilize management accounting information to adjust the probability rates in the models so that in every time interval they reflect the probability of finding a compound fit for clinical development as accurately as possible. For an investor, without access to this information, it may be possible to use official reports for the same purpose. If a project is just about to start, it might be rational to assume that no compounds will be generated during the first years, and that a certain starting time with a startup cost should apply. It might also be meaningful to add a delay to the probability time series so that the cash flows during one time period reflect the probability of generating a candidate drug during a later period. The knowledge that a relatively large amount of the cash flows will go to late preclinical development might give reason to increase the calculated *p*_*i*_s.

The question of how many compounds that are generated for clinical phase I trials is in reality not as straightforward as the models presented herein suggest. In drug discovery projects, usually a large number of highly reminiscent structures are synthesized, from which usually a number of compounds can be identified as good candidates for further development. However, only one or maybe two of these are generally chosen for clinical development. If a compound fails in clinical phase I, which is usually due to toxicity or other adverse effects, it is likely that other compounds with just minor changes to the structure will share the same problems. It may therefore be misjudged to look at each individual compound that could be considered for clinical development as a hit in the models presented. Rather, a structurally more distant molecule might be meaningful to take to the clinic. In fact, there exist mathematical methods to compute the chemical similarities of compounds, based on which it would be possible to implement filters to judge if a compound is truly ’novel’ in the sense of bringing it into clinical development, compared with previously failed compounds.

The models should therefore be regarded in instrumentalistic terms rather than as a reflection of how reality works. How many compounds that are considered fit for clinical development is dependent on how strict criteria that we apply in the selection process. With very liberal criteria, the probability of finding suitable compounds must be set high, but the lower success rate in the clinical development must be reflected in the parameters for probability in the clinical development.

At last we would like to mention that the practices for economic evaluation of biotechnology pure play enterprises is currently poorly developed. In a report from the management consulting firm McKinsey from 2000 based on interviews with 44 CEOs and business developers from representative pharmaceutical and biotechnology companies found that one third admitted not to employ any economically valid evaluation method. Among these, 21% used simple cost plus approaches and 12% simply made a guess (Moscho et al. 2000). This study argued that a reliable economic valuation is necessary in order to persuade financiators to invest in the enterprice and partners to form partnership agreements. Further on, it is potentially deleterious for all parties when deals are based on unrealistic expectations. It is also noteworthy that the pharmaceutical industry is in a crisis with declining productivity and increasing costs, which the major companies seek to counteract by in licensing only late projects in clinical development, shifting the major risk to small enterprises and start-ups, while on the other hand venture capital has become increasingly reluctant to fund the early projects. In an overall perspective these developments may not be cost-effective for the sake of public, for which there is still a very large unmet need for effective treatments of a large number of severe and disabling diseases. The development of methods for the rational evaluation of drug discovery is thus essential for a wealthy drug discovery sector to develop, to which end we hope that our proposed approaches will contribute.

## Conclusions

We have suggested three dedicated extensions to the net present value calculation for drug discovery projects. The process of setting parameters for the models and their overall utility has been discussed. We propose that the models shall be considered in the evaluation of early drug discovery endeavors for the future, and that their practical implementation for the purpose is a highly desired task to study.

## Abbreviations

ADMET: Absorbtion, distribution, metabolism, elimination and toxicology
CROs: Contract research organizations
*rNPV*: Risk-adjusted net present value
SARs: Structure-activity relationships.
